# The production of consumption: addressing the impact of mineral mining on tuberculosis in southern Africa

**DOI:** 10.1186/1744-8603-5-11

**Published:** 2009-09-29

**Authors:** Sanjay Basu, David Stuckler, Gregg Gonsalves, Mark Lurie

**Affiliations:** 1 Department of Medicine, University of California San Francisco, CA, USA; 2 Division of General Internal Medicine, San Francisco General Hospital, CA, USA; 3 University of Oxford, Department of Sociology, Oxford, Oxfordshire, UK; 4 Department of Ecology and Evolutionary Biology, Yale University, New Haven, CT, USA; 5 Department of Community Health, Brown University Medical School, Providence, RI, USA

## Abstract

**Background:**

Miners in southern Africa experience incident rates of tuberculosis up to ten times greater than the general population. Migration to and from mines may be amplifying tuberculosis epidemics in the general population.

**Discussion:**

Migration to and from mineral mines contributes to HIV risks and associated tuberculosis incidence. Health and safety conditions within mines also promote the risk of silicosis (a tuberculosis risk factor) and transmission of tuberculosis bacilli in close quarters. In the context of migration, current tuberculosis prevention and treatment strategies often fail to provide sufficient continuity of care to ensure appropriate tuberculosis detection and treatment. Reports from Lesotho and South Africa suggest that miners pose transmission risks to other household or community members as they travel home undetected or inadequately treated, particularly with drug-resistant forms of tuberculosis. Reducing risky exposures on the mines, enhancing the continuity of primary care services, and improving the enforcement of occupational health codes may mitigate the harmful association between mineral mining activities and tuberculosis incidence among affected communities.

**Summary:**

Tuberculosis incidence appears to be amplified by mineral mining operations in southern Africa. A number of immediately-available measures to improve continuity of care for miners, change recruitment and compensation practices, and reduce the primary risk of infection may critically mitigate the negative association between mineral mining and tuberculosis.

## Background

Miners in southern Africa have the highest tuberculosis incidence of any working population. Rates of TB are, to our knowledge, the greatest reported in the world: at least three times higher than in any country [1]. The effects of migration to and from the mines, the health and safety conditions within the mines, and the limitations to current tuberculosis prevention and treatment strategies offered to miners all contribute to the present tuberculosis burden among this population. The problem has recently been amplified by concerns about the emergence of drug resistant tuberculosis in southern Africa and the increasing frequency of travel between mines and rural communities. This article examines the relationships between mining and tuberculosis in southern Africa and describes current intervention options.

## Discussion

### Why are miners in southern Africa at high risk of tuberculosis?

The mining industry is among southern Africa's largest employers, particularly in the Republic of South Africa, where one of every ten employed men (at least 500,000 men) mines for gold, diamonds or other minerals [2,3]. The South African government reports the incidence of tuberculosis to be as high as 7,000 cases per 100,000 miners, about ten times higher than among the general population [4]. Very high rates of over 1,000 per 100,000 have been reported among miners in other southern African countries [5]. Miners have 3.6-fold greater odds of dying from tuberculosis than other workers in the region [6]. These risks of tuberculosis have evolved over the past century; at least as far back as 1903, mining was recognized as a risk factor for TB incidence and mortality [7]. A combination of environmental and occupational explanations have been proposed to explain the elevated risk of morbidity and mortality from tuberculosis among miners.

#### Silica exposure

Exposure to silica dust increases the risk of pulmonary tuberculosis, particularly among gold miners who drill through hard rock. Miners with the scarring lesions characteristic of silicosis--about 18% to 31% of goldminers in Botswana and South Africa--have about a three-fold increased risk of pulmonary tuberculosis compared with those without silicosis [8]. In a recent study of nearly 700 South African goldminers, 24% had silicosis; of the miners with silicosis, 44% had a history of tuberculosis, as compared to 26% among those without silicosis [8].

#### Occupational conditions

Living and working conditions are also a cause for concern. Mine shafts themselves are crowded and poorly-ventilated, but so are hostels where over a dozen men can share a small room [9]. These conditions are highly conducive to infection; the rate of recurrent tuberculosis in a recent South African prospective cohort of 600 miners was about 8 per 100 person-years (as opposed to half of that rate or less in the general population, [1]), with 69% of recurrent cases attributable to reinfection rather than relapse [10].

#### Migration and HIV

But occupational and environmental risks on mines apply to the mining sector in wealthy countries, just as in poor ones. What makes mining in southern Africa so dangerous that tuberculosis rates are far higher among African miners than in miners in the UK? While regulations are weaker in southern Africa, the companies owning the mines and determining typical occupational conditions are multi-national corporations. The problem is not simply one of differential occupational hazards, but of the social context for transmission and the interaction between miners and the rest of the population.

An extensive migration system throughout southern Africa was constructed over a century ago to facilitate the movement of workers to mines. The system, which until the early 1990's prohibited black workers from settling permanently in "whites-only" areas, created patterns of circular migration conducive to the spread of tuberculosis both on the mines and to rural areas from which men migrated [11]. Shantytowns developed around hostels, with alcoholism and prostitution proliferating around many. This corresponded to the spread of sexually-transmitted diseases [12,13].

HIV has rapidly spread among miners and their partners since the 1980's. The dramatic rise in HIV prevalence among miners (upwards of 30% in some cohorts) has been attributed in part to a subsequent increase in tuberculosis incidence among them [14]. According to one industry study, nearly one-third of new mineworkers without HIV will become infected within the first eighteen months of employment [15]. HIV increases the likelihood that a person infected with tuberculosis will progress to active disease, shortens survival times among co-infected individuals, and increases the likelihood of atypical tuberculosis manifestations that can be difficult to diagnose [16]. HIV-tuberculosis co-infection is particularly problematic for miners: HIV and silicosis multiplicatively increase the risk of tuberculosis, and tuberculosis incidence among HIV-positive silicotic miners is about 15 times higher than among HIV-negative miners without silicosis [17]. Migrants moving between their homes and the mines usually do not have continuous access to treatment, risking individual poor patient outcomes as well as the development and subsequent transmission of drug-resistant forms of disease.

### Public health consequences

#### Changing migration patterns

Since the relaxation of rules restricting movement under apartheid, miners are able to travel more frequently between mines and their home communities. In South Africa today, roughly 230,000 men migrate each year from other countries for mining jobs [18]. Over 50,000 men travel to South African mines from Lesotho, and 60% of them return home at least once per month; these individuals would normally travel home only once or twice per year in prior decades [19]. The circular migration pattern not only exposes people in low prevalence areas to migrants with a higher prevalence of HIV and tuberculosis, but also prevents continuity of care, adherence support, and consistent access to diagnostic facilities for migrating miners.

#### Rising drug-resistance

In a cross-sectional study of over 28,000 South African goldminers, 18% of 425 tuberculosis cases acquired multi-drug resistance, and a further 9% had already-resistant tuberculosis strains transmitted to them (primary resistance). Over 13% of cases had previously failed therapy [20]. Since August 2007, one-quarter of new multi-drug and extensively drug resistant tuberculosis cases in Lesotho were among miners or former miners [19].

#### Transmission to communities

**Figure 1:**
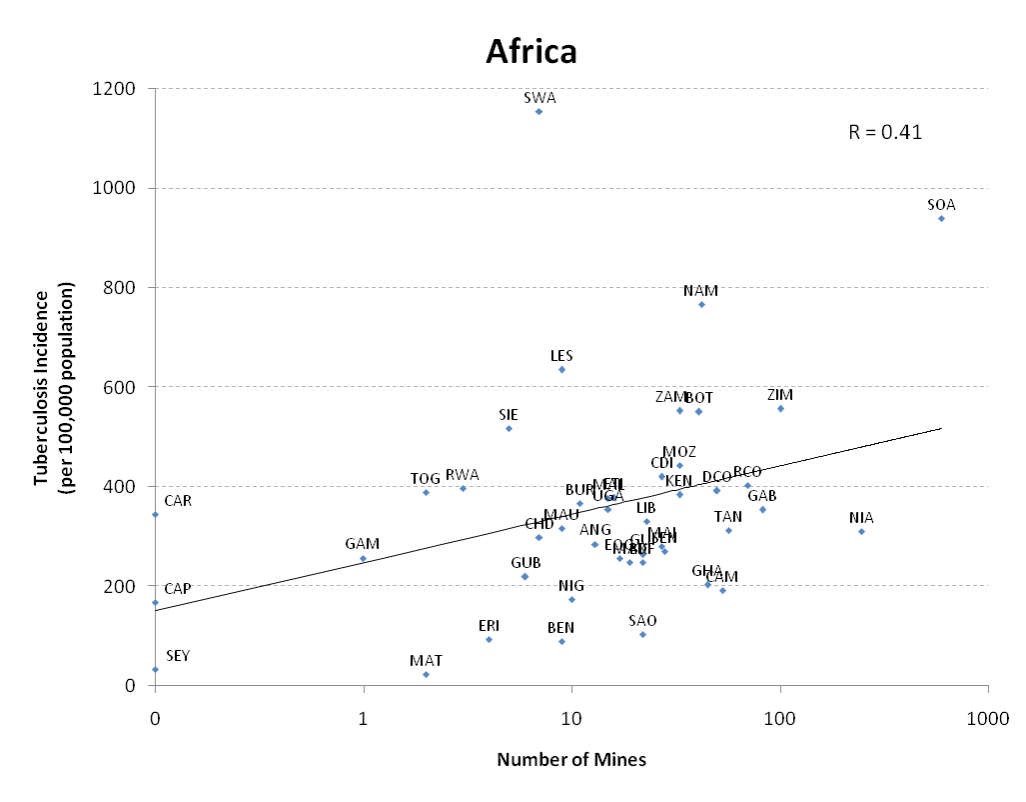
**The relationship of tuberculosis in the general population to the number of mines in the population among southern African nations (r = 0.41, p < 0.01)**.

Reports from communities affected by extensively drug-resistant tuberculosis suggest that miners pose transmission risks to other household or community members as they travel home undetected or inadequately treated [21]. The wave of new tuberculosis infections related to HIV is also being accompanied by significant secondary transmission of tuberculosis to HIV-uninfected persons [22]. Indeed, the number of mines in a population correlate strongly to the overall population's tuberculosis incidence (Fig. 1); while this suggests correlation and not causation, the finding indicates that the implications of mining for community-wide tuberculosis control requires further investigation.

### What is currently being done?

The mining industry has publicized its extensive system of tertiary care facilities, including over three dozen hospitals operated by several thousand healthcare staff. Miners typically receive annual physical examinations and X-rays to detect active tuberculosis, with laboratory confirmation of tuberculosis diagnosis. The industry also boasts full compliance with the World Health Organization directly-observed tuberculosis treatment, a silicosis prevention program involving dust control measures, and an antiretroviral treatment program [23].

But the mining industry is not required to report publicly its data on disease incidence and outcomes, and, in South Africa, the industry reports to a government agency that keeps its results secret. Some of the publicly-available information released by the mining industry about their medical operations appears to conflict with independent assessments and descriptive studies of miners' lives. Appendix 1 describes evidence revealing that (i) screening rates reported by mining companies are out of sync with autopsy data which reveal high rates of undetected active tuberculosis among miners, (ii) contract miners are excluded from care, and workers may be dismissed from work without follow-up treatment when showing symptoms of tuberculosis, and (iii) significant gaps in continuity of care and support for adherence to treatment exist in spite of extensive tertiary care facilities at some large mines. Although a review tool has become available for the mining sector to assess TB program performance (sponsored by the Department of Minerals and Energy in 2007), it has yet to be applied [24].

### What can be done?

At least three sets of interventions can be implemented to decrease tuberculosis incidence on the mines and connected communities.

#### Reducing risk

First, we must reduce the risk of tuberculosis among miners. The biological risks to miners in Africa should be no higher than the risks faced by miners in western countries. As the mining industry profits from a period of boom in mineral demand (in part due to gold demand and rising gold prices [2]), it has the capital that other industries are current lacking. Building hostel environments that reduce crowding and are designed with infection control in mind may mitigate transmission rates among workers. In an unprecedented step, one mining CEO abolished common residences and launched a housing scheme whereby miners could live with families. According to Deloitte, this led to the mining group experiencing one of the lowest HIV/AIDS growth rates in the industry, with clear benefits for reducing TB risk[15]

Silicosis can be addressed through improved enforcement of dust control regulations, yet most initiatives appear to be industry-determined, based on suggested guidelines, and ineffective. Indeed, the industry target for aggregate dust measurements (0.1 mg/m3 of aggregate dust) is not actually protective against silicosis [25]. Correcting this target is essential to preventing silicosis, as are follow-up screening and treatment services for former mineworkers, because silicosis develops over 15-20 years which means increased risk of TB could develop more than a decade after miners have stopped working [8].

HIV prevention approaches are based on 'information-dissemination', although there is no evidence that this approach, or alternative peer-education and condom distribution programs, have led to changes in sexual behavior among miners [26]. Structural interventions such as expanding access to family housing, addressing alcoholism, and ensuring appropriate viral suppression through antiretroviral treatment appear to be insufficiently administered in the absence of external supervision of the mining industry [27,28].

#### Expanding medical services

The mining industry must expand clinical services so that access is not concentrated exclusively around larger mines. Open access to data on case detection and outcomes is necessary to evaluate the quality of care. Well-coordinated tuberculosis detection and treatment programs have significantly lowered tuberculosis rates and stabilized multi-drug resistance in some mining populations [29]. However, even programs that have achieved and superseded conventional tuberculosis treatment targets (e.g., 85% of diagnosed subjects completing therapy) have not interrupted epidemic rates of new tuberculosis transmission among mineworkers [30]. Effectively treating cases of active tuberculosis will reduce incidence, but, given the delay between symptom onset and tuberculosis diagnosis, case detection and treatment alone may not be adequate to reduce the infectious periods of patients [31]. Isoniazid preventive therapy, which significantly lowers the likelihood that infected individuals will advance from latent to active tuberculosis, could help reduce the risk of infection [32]. Regardless of the implementation of preventive therapy, care must be provided without discrimination towards those with existing symptoms or prior diagnoses of TB, and the government enforcement of treatment standards is necessary to monitor mining healthcare operations to ensure non-discrimination.

#### Addressing migration

Finally, many of the medical effects of migration can be addressed through public health initiatives. At present, miners have medical records available at their industry-based sites of care, yet they sometimes also receive care at their clinics at home. Case reports suggest that many miners being treated for drug-resistant tuberculosis are unknowingly treated with inappropriate drug regimens, such as the use of drugs to which patients had likely previously developed resistance [19]. Allowing miners to carry standardized paper-based medical records with them on their trips home may make relevant treatment history more accessible to physicians. The mining industry coordinates an extensive recruiting system that finds workers in distant regions. This system can also be employed to provide the contact information of mining medical facilities to health posts in rural home regions, permitting the transfer of vital medical history when miners seek care.

The South African Development Community (SADC) trading group promotes cross-border employment throughout the southern African region; in turn, it bears some responsibility and possesses the regional authority to address the medical information challenges posed by cross-border TB management, including discrepancies in treatment regimens and the need for coherent and effective referral services for miners.

#### Coordination and Leadership

Overall, there is a critical absence of a focal point of government leadership and a clear delineation of responsibilities between different institutions. Several government ministries are involved in the management of TB in mining, including the Department of Minerals and Energy, the Department of Health, the Department of Labour. This results in dispersed responsibility and accountability for managing TB risks, and often means that progress in implementing effective risk reduction initiatives is slow and uneven. Given the clear demonstrated health impact of the mining sector on TB, and given that TB has been declared a public health emergency in the region, there is a strong case for the Department of Health in South Africa, and other health departments in the region, to assume a clear leadership role on this issue.

#### Challenges to implementation

Implementing these reforms will be met by considerable challenges. The mining industry currently lacks incentives to enhance tuberculosis prevention and treatment approaches, as the limited public information it disseminates suggests high-quality treatment facilities and extensive systems of care.

Yet, it is clear that the mining industry is not paying the full price of enhanced tuberculosis risk among its workers. Compensation for occupational disease remains difficult for miners' families to obtain, typically including only work shifts lost due to hospitalization, and requiring evidence of second degree tuberculosis or permanent lung damage (usually by demanding that families provide organ samples to a government bureau for investigation). Families typically report no compensation or delayed compensation, which contributes to poverty after the loss of the household breadwinner [19]. A 2005 report by DeLoitte suggested that the compensation fund used by mining companies is essentially bankrupt, and would require the industry to increase funding by 100-fold to fulfill miners' claims. The high financial cost of addressing TB is likely a key reason for the mining sector's reluctance to act decisively[15]

The South African parliament's recent passage of the Mines Health and Safety Amendment Bill imposes stricter penalties on mining companies that do not comply with required standards of health and safety. The law holds employers criminally liable for the loss of life, injury or ill-health that occurs as a result of neglect to take "all reasonable steps" to create a safe and healthy working environment [33]. Support from the public health community is needed to investigate independently the epidemiological claims of the mining industry and monitor miners' treatment, outcomes and compensation under this new legal system.

Enforcement of improved labor conditions and medical standards requires the active participation of miners' unions. Traditionally, mining unions have focused on occupational hazards in the form of accidents and trauma. While it is appropriate to have serious concerns about mining accidents, such accidents resulted in 199 deaths in 2006, TB, HIV and silicosis claimed at least 5,000 miners' lives that year [1,34]. Particularly after the end of apartheid in South Africa and the development of stronger unions in Botswana, unions have an opportunity to push government regulators into the enforcement of improved working standards. The unions must also address the issue of undocumented workers, who have been traditionally excluded from participation, but in recent years been growing as a proportion of mineworkers. These groups often have less job security and lower rates of access to compensation, as well as lack formal representation.

Government cooperation will be vital to address cross-border issues, particularly for cases of tuberculosis that are sent home without care, and new tuberculosis infections among family members; this requires that representatives recognize the health burdens of mining are falling on their constituents. A body of key stakeholders--miners' unions, medical providers, and government representatives--should assemble to determine how to enforce high standards of follow-up care and compensation, such that the burden of tuberculosis does not continue to be placed on labor-supplying communities., Government leadership and a clear delineation of responsibilities among government responsibilities is needed to move forward. In South Africa, various departments, ranging from the Minerals and Energy Department to the Department of Labour, could be directly or indirectly responsible for various components of the response to TB among miners. But the clear health impact of mining on TB, and the emergency-level of TB in the region, indicate that the Department of Health must be principal in taking a leadership role on this issue. Table 1 summarizes key areas for investigation and intervention.

**Table 1:** Recommendations for strengthening tuberculosis control strategies for miners and their communities

***Dimension***	***Issue***	***Recommendation***
**Healthcare**		

Primary Care	-Mines provide tertiary care for accidents and injuries but weak primary care	-Monitor adherence to treatment guidelines among medical providers
		-Evaluate and improve standards of living and safety on mines
		-Evaluate and implement isoniazid preventive therapy

Continuity of Care	-Mineworkers cross borders but care does not	-Provide a standardized set of patient-held medical records, and coordinate key care locations across borders
	-Doctors lack patient information needed to avoid breeding drug-resistant TB strains	-Key stakeholders, including mineworkers' representatives, should create a formal body to investigate and evaluate both within-country and cross-border TB management among migrants

**Recruitment**	-Contracts with loopholes	-Provide clear translation and communication of contract implications to workers, with union supervision
	-Poor literacy	-Include health benefits and protections for contract employees
		-Allow and encourage family housing and rights to permanent residence
		-Enforce and produce new laws for occupational safety

**Compensation**	-Former and informal barriers prevent mineworkers and their families from receiving compensation at all, or in a timely manner	-Avoid unnecessary restrictions and obstacles to qualify for compensation and receive it promptly
		-Create bodies of oversight to examine compensation system

## Conclusion

Mining continues vigorously in southern Africa, with companies from Australia, India, Russia, and the United Kingdom opening new mines throughout the region under the framework of recent foreign trading agreements [34]. As both the industry and its associated epidemics resurge in the region, the time is ripe for mining officials and public health practitioners to combat the avoidable risk of disease connected with mining. This region is not "resource-poor" but "resource-denied"; it is indeed ironic that the area's abundance of mineral resources has brought so much violence and disease. Mitigating the public health costs of mining is logistically complex, but potentially feasible with concerted effort. To engage the mining-tuberculosis relationship is a matter of health equity, as the fruits of mining have historically not been shared with those who occupy and mine the land, while the ill effects of mining have been disproportionately placed upon the poorest of communities.

## Summary

The effects of migration to and from mineral mines, health and safety conditions within mines, and limitations to current tuberculosis prevention and treatment strategies offered to miners all contribute to a high tuberculosis burden among this group of workers.

The circular migration of miners to and from their home communities poses the risk of increased tuberculosis exposure among home community members. Migration also prevents continuity of care, adherence support, and consistent access to diagnostic facilities among migrants.

Reducing risky exposures on the mines, enhancing the continuity of primary care services, and improving the enforcement of occupational health codes may mitigate the harmful association between mineral mining activities and tuberculosis incidence among affected communities.

## Abbreviations

TB: tuberculosis; HIV: human immunodeficiency virus.

## Competing interests

The authors declare that they have no competing interests.

## Authors' contributions

SB drafted the manuscript; DS, GG and ML provided data and assisted in revision of the manuscript.

## Appendix 1. Conflicts between Mining Company Reports and Independent Assessments

1) Medical programs are limited to larger mining houses and men who are currently employed. As increasing numbers of workers serve as contract employees, supplied by intermediary companies or otherwise not included in standard benefit scales, their entitlements to health benefits is limited [19].

2) In spite of mining company reports of annual or more frequent tuberculosis screening [23], a post-mortem study suggests that 40% of tuberculosis cases among miners are undetected during life [35].

3) Confirmation of appropriate treatment adherence also indicates that adherence to treatment is only 85% (rather than the reported 99.8%) among the 40% of patients who returned urine samples in a recent assessment. Thirty-five percent of patients' urine samples also contained tuberculosis drugs not recorded in their medical records [36].

4) In previous years, miners with tuberculosis were sent home without care, as mining companies avoided the costs of treating infected workers. While this practice is technically now illegal, recent case reports--particularly among foreign migrant workers infected with multi-drug or extensively drug resistant tuberculosis--suggest that the practice continues, producing a burden and risk for both miners and their labor-supplying communities [37].

5) Patients are treated through a hospital-based system, yet the extensive tertiary care facilities available on some mines lack an effectively-coordinated primary care backbone for treatment initiation and support [26]. Because this problem extends to HIV care as well, many miners have been found with advanced, untreated HIV disease, which misses the opportunity to decrease tuberculosis incidence through effective antiretroviral therapy [19,38].
